# Improving Soybean Development and Grain Yield by Complementary Inoculation with Growth-Promoting Bacteria *Azospirillum*, *Pseudomonas*, *Priestia*, and *Bacillus*

**DOI:** 10.3390/plants14030402

**Published:** 2025-01-29

**Authors:** Robélio Leandro Marchão, Gustavo Cassiano da Silva, Solange Rocha Monteiro de Andrade, Fábio Bueno dos Reis Junior, Márcio Pereira de Barros Júnior, Richard Hemanwel Haphonsso, Arminda Moreira de Carvalho

**Affiliations:** 1 Embrapa Cerrados, BR 020, Km 18, Caixa Postal 08223, Planaltina 73.310-970, DF, Brazil; gustavo.cassred@hotmail.com (G.C.d.S.); solange.andrade@embrapa.br (S.R.M.d.A.); fabio.reis@embrapa.br (F.B.d.R.J.); arminda.carvalho@embrapa.br (A.M.d.C.); 2 Department of Cell Biology, University of Brasilia, Campus Darcy Ribeiro, Caixa Postal 131, Asa Norte 70.910-970, DF, Brazil; marciojr.biologia@gmail.com; 3 Federal Institute of Brasília, Rodovia DF 128–Km 21 S/N Zona Rural, Caixa Postal 002, Planaltina 73.380-900, DF, Brazil; richardhemanwel@gmail.com

**Keywords:** sustainable intensification, bioinputs, regenerative agriculture, *Glycine max* (L.) Merrill

## Abstract

Bioinputs are natural products applied to crops that contribute to more sustainable agriculture by boosting yields and reducing environmental impacts. In Brazil, the use of bioinputs such as *Bradyrhizobium* in soybean has been consolidated, but the expansion of on-farm bioinput production is currently initiating a new revolution. Furthermore, applications of bioinputs to cash crops in Brazil have shed light on the great potential of such growth-promoting microorganisms (GMPs) to improve nutrient uptake and increase productivity. This study explores the effect of the complementary inoculation with growth-promoting bacteria of post-emergence soybean, previously inoculated with *Bradyrhizobium* spp. Five treatments with growth-promoting bacteria were evaluated: T1—Control (no inoculation); T2—*Azospirillum brasilense*; T3—*Pseudomonas fluorescens* and *Azospirillum brasilense*; T4—*Priestia aryabhattai*, *Bacillus haynesii*, and *Bacillus circulans*; and T5—*Priestia megaterium* and *Bacillus subtilis*. In comparison with the control, all treatments with growth-promoting bacteria of the genera *Azospirillum*, *Pseudomonas*, *Priestia*, and *Bacillus*, applied after soybean emergence, induced 4–7% higher grain yields. Co-inoculation with *Priestia megaterium* and *Bacillus subtilis* (treatment T5) resulted in a higher 1000-grain weight, while *Priestia aryabhattai*, *Bacillus haynesii*, and *Bacillus circulans* (treatment T4) increased the number of pods and shoot dry weight. Our conclusion is that bioinputs increase soybean productivity and make agriculture more sustainable and efficient.

## 1. Introduction

Soybean (*Glycine max* (L.)) is one of the main agricultural crops in the world and plays a key role in food, feed, and biofuel production. Brazil is the largest producer, where soybean is grown on approximately 45.9 million hectares, with an output of over 147 million tons in the 2023/2024 growing season [1]. Efforts have been concentrated to find more effective alternative inputs for soybean production, with minimal environmental impact [2].

Brazilian agriculture attracts attention with the use of bioinputs, particularly regarding soybean, where the biological fixation of nitrogen is stimulated by seed inoculation with *Bradyrhizobium* ssp. This practice reduces the need for chemical fertilizers and contributes to more sustainable agricultural management. Currently, a second agricultural revolution is going on in the country, mainly by the use of plant-growth-promoting microorganisms. These bioinputs, based on a set of bacterial and fungal species, provide several benefits, such as increased root growth, phosphorus solubilization, resistance to environmental stresses, and improved plant physiology. They can optimize nutrient uptake and increase plant productivity, which makes agriculture more efficient and sustainable.

In this context, the use of bioinputs and their large-scale commercial use are of particular interest. This trend has been intensified in Brazil, within an ecological transition strategy in agriculture [3]. The transition is being driven by the installation of on-farm biofactories, which allows the multiplication and local reproduction of bioinputs [4]. This facilitates the producers’ access to these sustainable solutions, promoting greater autonomy, efficiency, and savings in agricultural production.

These products are being widely used by small- and medium-sized rural producers in several developed countries [5]. The global bioinput market was estimated at USD 8.8 billion in 2019 and is expected to grow at a CAGR (Compound Annual Growth Rate) of 13.6% to reach a value of USD 18.9 billion by 2025 [6]. Bioinputs are formulations of elements of biological origin that can include microorganisms, plant extracts, and organic compounds, among others, which promote plant growth, increase resistance to pests and diseases, and improve the soil quality [7].

For soybean, bioinputs have several environmental benefits that make crops safer and more resilient. These inputs can play an important role in reducing the dependence on agrochemicals (fertilizers and pesticides), in conserving natural resources, and in mitigating environmental impacts on agriculture [8]. Therefore, it is imperative to conduct further research on the inclusion of bioinputs in agriculture, and clear public policies must be created to stimulate and promote their use in the tropics. Some of the main bioinputs are inoculants containing beneficial microorganisms, e.g., a number of genera of bacteria such as *Bradyrhizobium*, *Bacillus*, *Priestia*, *Pseudomonas*, and *Azospirillum*, known as plant growth promoters. These microorganisms have beneficial effects on plants and colonize the rhizosphere as well, which improves their performance under adverse environmental conditions [9,10].

The inoculation of soybean seeds with *Bradyrhizobium* spp. promotes biological nitrogen fixation in the soil. With this technique, up to 94% of the plant nitrogen demand can be met, which corresponds to approximately 300 kg N ha^−1^ per hectare. These inputs reduce the dependence on synthetic nitrogen fertilizers, resulting in savings of approximately USD 3.2 billion [11].

Inoculants that contain *Bacillus subtilis* and *Priestia megaterium* strains produce different organic acids capable of solubilizing phosphates combined with calcium, aluminum, and iron in the soil, and make P readily available to plants. These two strains were isolated in different agricultural areas in the country, where cereal cultivation prevails [12]. Another phosphorus-solubilizing genus in the soil is *Pseudomonas*, which is a quite common soil bacterium. The bacterium *Priestia aryabhattai*, native to the Caatinga biome [13], can improve plant resistance to abiotic stresses, e.g., drought, salinity, and extreme temperatures [14]. These advantages are particularly relevant in the context of adaptation to climate change, with its increasing challenges for plants. These microorganisms are capable of hydrating roots and influencing plant physiology, enabling plants to respond better to water scarcity.

*Azospirillum* spp. are free-living soil bacteria that have the capacity to fix biological nitrogen symbiotically with plants. However, they are mainly known for other mechanisms, such as the production of phytohormones (e.g., indole-3-acetic acid—IAA) that influence plant growth and development, especially of the roots, thus optimizing the efficiency of water and nutrient use. These bacteria are recommended for inoculation of a wide range of species, particularly of the *Poaceae* family, and are commonly used in maize cultivation [15]. For legumes, e.g., bean and soybean, it is recommended to co-inoculate *Azospirillum* spp. with *Bradyrhizobium* sp. to improve crop performance, in an approach to meet the current demand for sustainable and regenerative agriculture [16]. In view of the benefits of inoculants, the use of this type of bioinput is on the rise in Brazil [3]. With a view to enabling producers to optimize the use of this technology, the development of new technologies and the definition of recommendations for the main crops in the country are vitally important. In this context, the objective of this study was to evaluate the effect of the complementary application of growth-promoting bacteria of the genera *Azospirillum* spp., *Pseudomonas* spp., *Priestia* spp., and *Bacillus* spp. on emerged soybean crops, previously inoculated with *Bradyrhizobium* spp., and the impacts of this combination on promoting soybean growth, development, and yield.

## 2. Results

### 2.1. Chlorophyll Content Index (SPAD) in Response to Bioinputs

**Figure 1:**
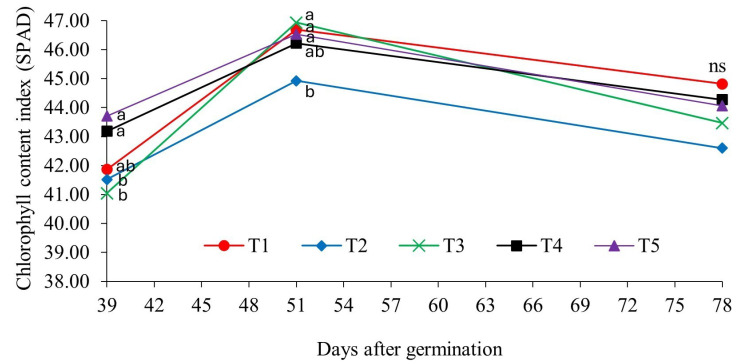
Chlorophyll content index in response to bioinputs (T1—Control; T2—*A. brasilense* strains Ab-V5 and Ab-V6; T3—*P. fluorescens* strain CNPSo 2719 and *A. brasilense* strains CNPSo 2083 and CNPSo 2084; T4—*P. aryabhattai*, *B. haynesii*, and *B. circulans*; and T5—*P. megaterium* strain BRM 119 and *B. subtilis* strain BRM 2084), in growing season 2023/2024. Planaltina/DF. Means followed by the same letter in a row do not differ at 5% probability by Duncan’s test.

The chlorophyll content index (soil–plant analysis development—SPAD value) of soybean was influenced by the presence of *Bradyrhizobium elkanii* associated with growth-promoting microorganisms. In the vegetative stages of the crop, the index differed significantly between the treatments, except in reproductive stage R6 (full grain filling—78 days after germination) (Figure 1).

In reproductive stage R3 (early pod formation—39 days after germination), chlorophyll contents were highest in treatment T4 (*P. aryabhattai*; *B. haynesii*; *B. circulans*) and T5 (*P. megaterium*—BRM 119; *B. subtilis*—BRM 2084), i.e., both included representatives of the genera *Bacillus* and *Priestia*.

In phenological stage R4 (pod filling—39 days after germination), mainly, plants treated with *A. brasilense* strains (Ab-V5) and Ab-V6 (T2) had a lower SPAD index than in the other treatments. The SPAD index decreased with advancing phenological stages (78 days after germination), as the physiological maturity stage of the plant passed and leaf senescence set in, which alters the chlorophyll level. The results indicate that the influence of complementary inoculation with growth-promoting bacteria on chlorophyll levels in soybean varies depending on the microorganisms used and the different phenological stages of the crop. The effect of the bacteria is not uniform and may be more or less pronounced, depending on the stage of plant development and specific interactions with the microorganisms.

### 2.2. Development of Soybean Plants in Response to Bioinputs

**Figure 2:**
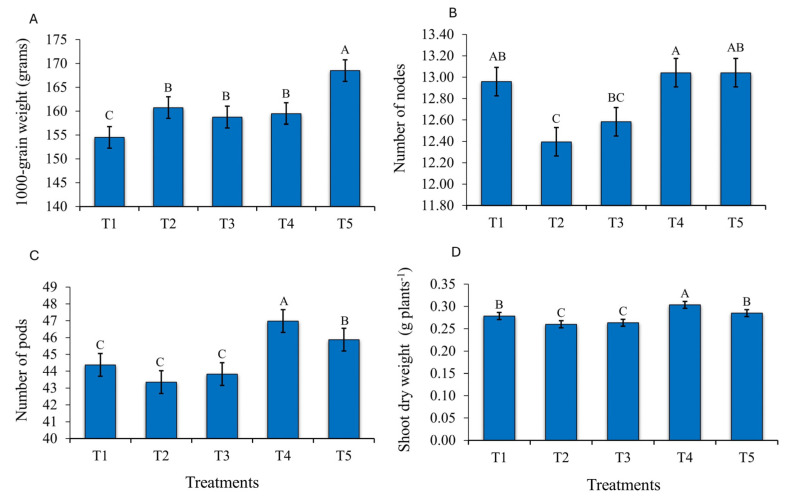
Soybean performance in response to bioinputs. 1000-grain weight (**A**), number of nods (**B**), number of pods (**C**), and shoot dry weight (**D**) in response to different bioinputs (T1—Control; T2—*A. brasilense* strains Ab-V5 and Ab-V6; T3—*P. fluorescens* strain CNPSo 2719 and *A. brasilense* strains CNPSo 2083 and CNPSo 2084; T4—*P. aryabhattai*, *B. haynesii*, and *B. circulans*; and T5—*P. megaterium* strain BRM 119 and *B. subtilis* strain BRM 2084), in growing season 2023/2024. Planaltina/DF. Means followed by the same letter do not differ at 5% probability by Duncan’s test.

The treatments with bioproducts containing *Bacillus* and *Priestia* increased the mean values of agronomic traits, namely of 1000-grain weight, number of nodes, number of pods, and shoot dry weight (Figure 2). These results reinforce the positive effect of *Bacillus* and *Priestia* application on soybean growth and agronomic performance. For the variable 1000-grain weight, inoculation with *P. megaterium* and *B. subtilis* (T5) resulted in a mean weight increase of 8.49% over the control (Figure 2A). For the variable plant height, no statistical differences were observed between the evaluated treatments. The plants were tallest (84.06 cm) in the treatment with *P. aryabhattai*, *B. haynesii*, and *B. circulans* (T4), compared with the control (81.81 cm).

In the soybean plants inoculated with T4 and with *P. megaterium* and *B. subtilis* (T5), the variable number of nodes increased. However, this treatment did not differ from the control (check without complementary inoculation with growth-promoting microorganisms) (Figure 2C).

The number of pods increased when the plants were inoculated with *P. aryabhattai*, *B. haynesii*, and *B. circulans* (T4), with an increase from 44 pods per plant in the control to 47, which reinforces the positive influence of these bacteria on plant development (Figure 2D).

Shoot dry weight was significantly higher in plants inoculated with *P. aryabhattai*, *B. haynesii*, and *B. circulans*, with an increase from 0.28 g plants^−1^ in the control (T1) to 0.30 g plants^−1^ in T4, i.e., an equivalent increase of 500 kg ha^−1^ (Figure 2D).

### 2.3. Soybean Yield in Response to Bioinputs

**Figure 3:**
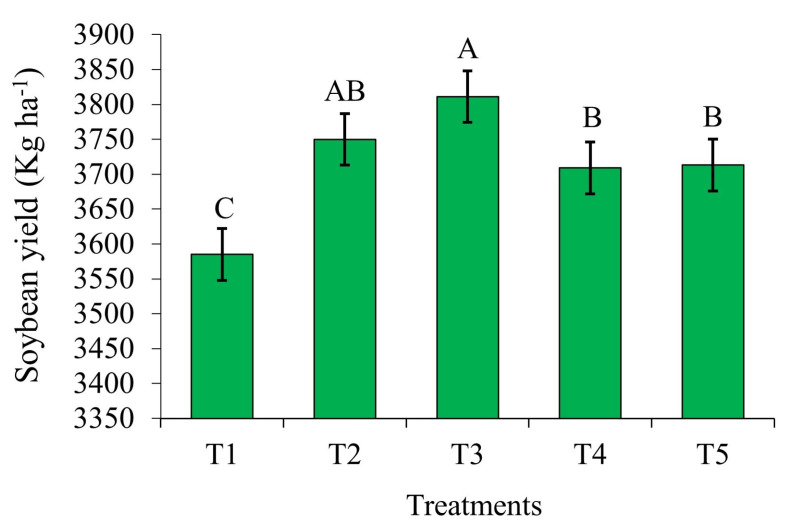
Soybean yield (kg ha^−1^), in response to different bioinputs (T1—Control; T2—*A. brasilense* strains Ab-V5 and Ab-V6; T3—*P. fluorescens* strain CNPSo 2719 and *A. brasilense* strains CNPSo 2083 and CNPSo 2084; T4—*P. aryabhattai*, *B. haynesii*, and *B. circulans*; and T5—*P. megaterium* strain BRM 119 and *B. subtilis* strain BRM 2084), in growing season 2023/2024. Planaltina/DF. Means followed by the same letter do not differ at 5% probability by Duncan’s test.

All products containing growth-promoting bacteria of the genera *Azospirillum*, *Pseudomonas*, *Priestia*, and *Bacillus*, when applied to emerged soybean, increased grain yield, compared to the control (Figure 3). In T2 and T3, with *Azospirillum* and *Priestia* inoculation, grain yields were significantly higher than in the other treatments.

Treatment T2, inoculated after emergence with *A. brasilense* strains Ab-V5 and Ab-V6, produced 3750 kg ha^−1^, which represents an increase of 165 kg ha^−1^ per hectare over the control. On the other hand, T3, treated with *P. fluorescens* strain CNPSo 2719 and *A. brasilense*s strains CNPSo 2083 and CNPSo 2084, yielded 3811 kg ha^−1^, i.e., an increase of 226 kg ha^−1^ per hectare in relation to the control.

## 3. Discussion

### 3.1. Influence of Growth-Promoting Bacteria on the Chlorophyll Content Index (SPAD) in Soybean

Inoculation with bacteria that promote phosphorus uptake increases chlorophyll levels of inoculated plants [17]. This is the case with bacteria of the genera *Bacillus* and *Priestia*, which are classified as solubilizers of inorganic phosphates [18,19]. They are capable of releasing phosphate adsorbed in the soil and making it available to plants. This reduces the need for future fertilization, prevents excessive phosphorus accumulation, and minimizes economic and environmental impacts, such as pollution by eutrophication [20].

Chlorophyll pigments are fundamental indicators to be considered in cultivation systems, since these molecules are responsible for photosynthesis, the process by which plants convert sunlight into chemical energy [21]. Chlorophyll levels are generally positively related to the plant nitrogen content. Although phosphorus is not a component of the chlorophyll molecule, it provides energy for the active uptake of nitrogen (N), which is a constituent of the porphyrin ring of the molecule, as described by Taiz and Zeiger [22].

Therefore, for soybean cultivation, inoculation with *Brayrhizobium elkanii* and the complementary application of phosphorus solubilizers of the genera *Bacillus* and *Priestia* provide higher chlorophyll levels in the initial phase of soybean reproduction (R3). In a study on soybean development, Costa et al. [23] observed that chlorophyll, an indicator of plant adaptability and growth in different environments, responded positively to higher rates of a *B. subtilis*-based product, in comparison with the control treatment, both in the early development stage, at 15 days after sowing (DASs), and 45 DASs, and for both tested cultivars. Lima et al. [24] evaluated the yield of maize inoculated with *B. subtilis*, with and without nitrogen fertilization, and found higher chlorophyll levels in the treatments with inoculation than those without inoculation. Similarly, Costa et al. [23] observed an increase in the SPAD chlorophyll index in soybean leaves in response to higher *B. subtilis* rates.

### 3.2. Soybean Growth and Development After Application of Growth-Promoting Microorganisms

The use of beneficial microorganisms in cultivation systems contributes to sustainable agriculture, resulting in improved crop growth, development, and grain yield, without damaging the environment. Inoculation with bacteria of the genera *P. megaterium* and *B. subtilis* can result in more robust and heavier grains, contributing to an increase in 1000-grain weight. Similar results were found by Lima and Busso [25], who evaluated the use of *P. megaterium* and *B. subtilis* in the treatment of hybrid maize seeds in the second crop. They observed increases in 1000-grain weight in response to rising rates of these microorganisms.

Growth-promoting bacteria also have beneficial effects on plants through different mechanisms of action, whether direct (biological nitrogen fixation, phosphate solubilization, phytohormone production) or indirect (siderophore and biofilm production) [26]. These bacteria can produce phytohormones such as auxins, cytokinins, and gibberellins, which stimulate cell division and stem elongation, favoring the formation of new nodes. This effect can be observed in response to inoculation with *P. aryabhattai*, *B. haynesii*, and *B. circulans* (T4) and *P. megaterium* and *B. subtilis* (T5), evaluated in this study.

In an evaluation of soybean seeds inoculated with growth-promoting microorganisms, Mattos [27] observed that only *B. subtilis* promoted an increase in the number of nodes per plant, from the control without inoculation, with 16.20 nodes, to a total of 17.70 productive nodes on inoculated plants.

In the evaluation of soybean growth and development in response to bioinputs, monitoring the number of pods is of utmost importance. These data can contribute to make production management more efficient, allowing more accurate decisions and, consequently, better agronomic results. It is worth mentioning that although the number of pods per plant is an important variable, it can vary depending on a variety of factors, such as genetics, environment, biotic and abiotic factors, and management practices.

The results indicated that inoculation with bacteria of the genera *P. aryabhattai*, *B. haynesii*, and *B. circulans* was the most efficient in increasing the number of pods in soybean. Contrary results were reported by Silvestrine et al. [28], who observed no significant effect on the number of pods per soybean plant in response to phosphate fertilization and inoculation with *Bacillus* sp. in the field.

Another important variable in crop development is shoot dry weight, which is an indicator of the plant dry weight and reflects the total amount of organic matter a plant produces. The increase in dry weight in plants treated with complementary inoculation with microorganisms (*P. aryabhattai*, *B. haynesii*, *B. circulans*) indicated a more robust growth and development than of the control plants, inoculated only with *Bradyrhizobium elkanii* at planting. In addition, the higher dry weight production can be reflected by a higher straw dry weight, which is important for soil cover and protection against erosion.

*Priestia aryabhattai*, *B. haynesii*, and *B. circulans* seem to be promising for use in agriculture, due to the vast range of benefits these bacteria can confer to plants, ranging from increased resistance to abiotic stresses, such as drought, to nutrient availability [29]. Park et al. [30] found that a strain of *P. aryabhattai* increased soybean and rice growth significantly. By scanning microscopy, they also observed that the strain colonized the roots successfully within two days after inoculation, which resulted in a greater shoot length than in the control treatment.

### 3.3. Effect of Supplemental Application of Growth-Promoting Bacteria on Soybean Grain Yield

Complementary inoculation is an advantageous alternative for producers, as it increases crop yield and also contributes to the sustainability of the system. This technique improves the efficiency of nitrogen fixation by plants and reduces the need for synthetic nitrogen fertilizers. In this way, not only can input costs be reduced, but the negative impacts on the environment associated with an excessive use of chemical fertilizers, such as soil and water pollution, can also be minimized.

The results of the combined use of *B. elkanii* and *A. brasilense* in soybean are promising according to Benintende et al. [31], in that mean yield increases of 7.7% have been reported [16]. In two growing seasons and at four locations (Londrina and Ponta Grossa in Paraná, where populations of *Bradyrhizobium* spp. were already established, and Rio Verde and Cachoeira Dourada in Goiás, without previous populations of these bacteria), the same authors observed higher yields at all locations in response to this combination, compared to *Bradyrhizobium* spp. inoculation only and equal to fertilization with 200 kg N ha^−1^ in the treatment without inoculants.

## 4. Materials and Methods

### 4.1. Experimental Area

**Figure 4:**
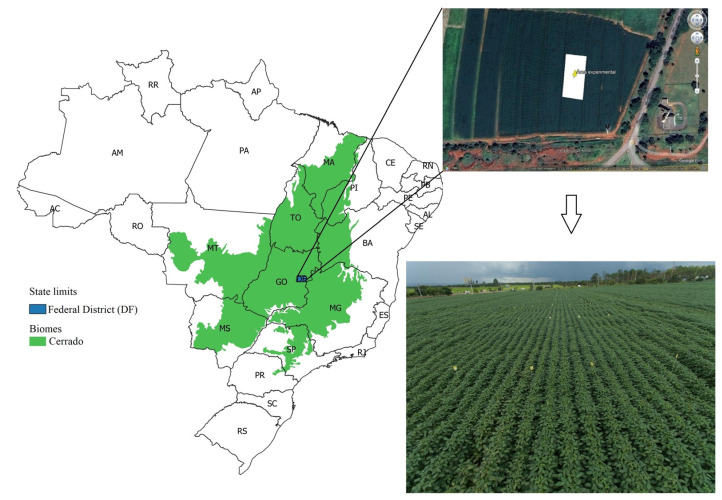
Location of the experimental area.

This study was carried out in the experimental area of Embrapa Cerrados, in Planaltina, Distrito Federal (15°36′38.82″ S, 47°42′13.63″ W; 980 m asl.). Soybean was planted on 1 December 2023 and harvested on 22 March 2024 (Figure 4).

**Figure 5:**
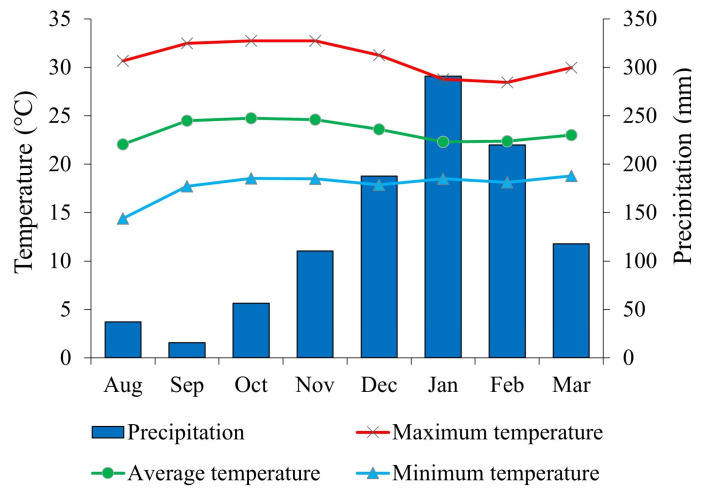
Rainfall, maximum temperature, and mean and minimum temperature during the experimental period of the 2023/2024 growing season.

In 2023, cumulative rainfall at the meteorological station near the experimental area was 791 mm. During the soybean cycle (December 2023 to 22 March 2024), cumulative rainfall was 773 mm (Figure 2). The maximum, mean, and minimum temperatures in the same period were 30.88, 23.39, and 17.80 °C, respectively (Figure 5). The regional climate was classified as Aw (Köppen’s classification). The soil was identified as a Red Latosol with a clayey texture. Prior to the installation of the experiment, soil chemical and physical properties (0–20 and 20–40 cm layers) were determined (Table 1).

According to the methods proposed by Teixeira et al. [32], the following soil chemical attributes were determined: pH (H_2_O), soil organic matter (SOM), K^+^, Ca^2+^, Mg^2+^, P, and potential acidity (H + Al^3+^). Based on the results of the chemical analysis, the following were calculated: cation exchange capacity at pH 7 (T) and base saturation (V%).

### 4.2. Experimental Design

The experiment was arranged in a randomized block design, with four replications. The five treatments consisted of formulations of commercial products with different bacteria, namely: T1—Control (without inoculation); T2—*Azospirillum brasilense* strains Ab-V5 and Ab-V6; T3—*Pseudomonas fluorescens* strain CNPSo 2719 and *Azospirillum brasilense* strains CNPSo 2083 and CNPSo 2084; T4—*Priestia aryabhattai* strain CBMAI 1120, *Bacillus haynesii* strain CCT 7926, and *Bacillus circulans* strain CCT 0026; and T5—*Priestia megaterium* strain BRM 119 and *Bacillus subtilis* strain BRM 2084. The experimental area of 600 m^2^ (60 × 10 m) was divided into 20 plots.

### 4.3. Management and Application Time

In all treatments, the seeds were inoculated with a commercial product containing the *Brayrhizobium elkanii* bacteria, composed of two strains of nitrogen-fixing bacteria (SEMIA 5019—50% and SEMIA 587—50%). A rate of 200 mL was used for 100 kg of seeds, inoculated immediately before sowing, using an electric concrete mixer.

The *Brayrhizobium elkanii* inoculant used in this study was a commercial product in liquid form, with a minimum concentration of 1 × 10^10^ CFU/mL, as recommended for soybean cultivation, according to the official protocol of the Ministry of Agriculture, Livestock and Supply (MAPA, 2010). Storage conditions according to the recommendation for the bacterial culture were ensured.

At sowing, all soybean seeds, cv. BRS 7080 IPRO, were inoculated with the strains SEMIA 5019 and SEMIA 587 of *Bradyrhizobium elkanii*. When the crop reached stage V5/V6, supplementary bio-inputs were applied with a CO_2_ pressurized backpack sprayer at a constant boom sprayer pressure of 3.0 kg cm^−2^. The spraying was performed with a five-nozzle boom that covered all five rows of a plot, applied at a height of 0.50 m above the target. The rates of each commercial product were defined according to the manufacturers’ recommendations for soybean. Although the products met the expiration date, the bacterial colonies were counted as described by Bettiol et al. [33].

The plant-growth-promoting bacteria were purchased from commercial companies and the rates were defined as recommended by the manufacturers. Applications were performed after soybean emergence, between vegetative stages V5 (fifth node—fourth fully developed trifoliate leaf) and V6 (sixth node—fully developed fifth trifoliate leaf), as shown in Table 2.

Before planting, fertilization (150 kg ha^−1^ of potassium chloride (K_2_O) and 200 kg ha^−1^ of monoammonium phosphate (MAP)) was broadcast in the planting row.

The phytosanitary management consisted of specific products applied during the crop cycle, according to the technical recommendations for soybean. Herbicide was applied once after emergence, and insecticides and fungicides twice. The following commercial products were applied: imazethapyr (100 g a.i./L) at a rate of 0.5 L ha^−1^; glyphosate (480 g a.i./L) at 3 L ha^−1^; and clethodim (240 g a.i./L) at a rate of 0.3 L ha^−1^. The insecticides thiamethoxam + lambda-cyhalothrin (141 + 106 g a.i./L) were applied at a rate of 0.3 L ha^−1^; pyriproxyfen (100 g a.i./L) at 0.25 L ha^−1^; chlorpyrifos (480 g a.i./L) at 1 L ha^−1^; and acetamiprid (150 g a.i/Kg) at 0.15 Kg ha^−1^; and the fungicides trifloxystrobin + tebuconazole (100 + 200 g a.i./L) were applied at 0.75 L ha^−1^ and bixafem + prothioconazole + trifloxystrobin (125 + 175 + 150 g a.i./L) at 0.5 L ha^−1^.

### 4.4. Chlorophyll Index Determination (SPAD)

The chlorophyll index was determined in reproductive stages R3 (beginning of pod formation), R4 (pod filling), and R6 (full grain filling), with a portable electronic chlorophyll meter (SPAD-502 Plus, Konica Minolta, Osaka, Japan). This device determines the relative amount of chlorophyll by measuring the absorbance at two wavelengths (650 nm—red light and 940 nm—infrared light) [34].

The third trifoliate of the upper third of the plant was used for the measurements. Six measurements per trifoliate were made to compute the mean values of each plant. Per treatment and replication, five randomly selected uniform plants were evaluated.

### 4.5. Determination of Yield and Grain Yield Components

Grain yield was evaluated by manually harvesting the plants growing in 2 m of the three central rows of each plot. In addition to yield, the following yield components were measured: plant height, number of nodes, number of pods, shoot dry weight, and 1000 g weight. Grain yield was estimated after mechanical threshing of the pods and weighing the grain of the areas considered for the evolution of each plot, assuming a moisture content of 13% [35].

### 4.6. Statistical Analysis

The data obtained were subjected to analysis of variance (ANOVA) and, in the case of significance of the treatments, the means were compared by Duncan’s test (*p* < 0.05). Statistical analyses were carried out using R software version 4.3.3 (R CORE TEAM, 2024).

## 5. Conclusions

All products containing growth-promoting bacteria of the genera *Azospirillum*, *Pseudomonas*, *Priestia*, and *Bacillus* when applied to emerged soybean promoted a higher grain yield compared to the control. This demonstrates the potential of these bacteria to improve crop yields. Among the evaluated commercial formulations, those containing *Azospirillum brasilense* induced the highest gains in grain yield. In terms of 1000 g weight, number of pods, and shoot dry matter, the bacterial species *P. aryabhattai*, *B. haynesii*, *B. circulans*, *P. megaterium*, and *B. subtilis* achieved the best results. However, plant height was not affected by the evaluated bioproducts, indicating that the beneficial effects were concentrated on characteristics directly related to grain yield. These results reinforce the potential use of these growth-promoting bacteria as an effective strategy by which soybean yield can be increased, without negatively affecting other agronomic aspects of the crop.

## Figures and Tables

**Table 1:** Soil chemical properties (0–20 layer) in 2023, prior to the experiment.

Layer	pH (H_2_O)	P	K^+^	Al^3+^	Ca^2+^	Mg^2+^	H^+^ + Al^3+^	T	V	SOM
(cm)		mg/dm^−3^	mg/dm^−3^	cmol_c_/dm^3^	cmol_c_/dm^3^	cmol_c_/dm^3^	cmol_c_/dm^3^	cmol_c_/dm^3^	%	dag kg^−1^
0–20	5.67	8.06	16.66	0.01	3.44	1.59	5.87	11.21	47.71	3.10

P: phosphorus (mg/L), K^+^: potassium (mg/L), Al^3+^: aluminum (cmol_c_/dm^3^), Ca^2+^: calcium (cmol_c_/dm^3^), Mg^2+^: magnesium (cmol_c_/dm^3^), H^+^ + Al^3+^: potential acidity (cmol_c_/dm^3^), T: cation exchange capacity at pH 7 (cmol_c_/dm^3^), V: base saturation (%), SOM: soil organic matter.

**Table 2:** Manufacturer, commercial products, treatments, growth-promoting bacteria, rate of commercial product per hectare, time of application, and concentration of active ingredient in a soybean field with complementary application of bioinputs.

Manufacturer	Commercial Products	Treatment	Strains	Rate	Application Time	Concentration
-	-	T1	No application	0	0	0
Biotrop	Azotrop	T2	*Azospirillum brasilense*s strains Ab-V5 and Ab-V6.	300 mL ha^−1^	V5/V6	2 × 10^8^ CFU/mL
Biotrop	Biopasto	T3	*Pseudomonas fluorescens* strain CNPSo 2719; *Azospirillum brasilense*s strains CNPSo 2083 and CNPSo 2084.	750 mL ha^−1^	V5/V6	1 × 10⁸ CFU/mL + 2 × 10⁸ CFU/mL
Biotrop	Bioasis	T4	*Priestia aryabhattai* strain CBMAI 1120;* Bacillus haynesii* strain CCT 7926;* Bacillus circulans* strain CCT 0026.	200 mL ha^−1^	V5/V6	2.1 × 10^9^ CFU/mL + 3.0 × 10^8^ CFU/mL + 8.8 × 10^8^ CFU/mL.
Simbiose	BiomaPhos	T5	*Priestia megaterium* strain BRM 119; *Bacillus subtilis* strain BRM 2084.	250 mL ha^−1^	V5/V6	4 × 10^9^ CFU/mL

T1—Control (without inoculation); T2—*Azospirillum brasilense* strains Ab-V5 and Ab-V6; T3—*Pseudomonas fluorescens* strain CNPSo 2719 and *Azospirillum brasilense* strains CNPSo 2083 and CNPSo 2084; T4—*Priestia aryabhattai* strain CBMAI 1120, *Bacillus haynesii* strain CCT 7926, and *Bacillus circulans* strain CCT 0026; and T5—*Priestia megaterium* strain BRM 119 and *Bacillus subtilis* strain BRM 2084. The mention of brands does not suggest a recommendation by the authors.

## Data Availability

The data presented in this study are available on request from the corresponding author.
